# The *Tinkerbell* (*Tink*) Mutation Identifies the Dual-Specificity MAPK Phosphatase INDOLE-3-BUTYRIC ACID-RESPONSE5 (IBR5) as a Novel Regulator of Organ Size in Arabidopsis

**DOI:** 10.1371/journal.pone.0131103

**Published:** 2015-07-06

**Authors:** Kim L. Johnson, Sascha Ramm, Christian Kappel, Sally Ward, Ottoline Leyser, Tomoaki Sakamoto, Tetsuya Kurata, Michael W. Bevan, Michael Lenhard

**Affiliations:** 1 ARC Centre of Excellence in Plant Cell Walls, School of Botany, University of Melbourne, Royal Parade, Parkville, Victoria, 3010, Australia; 2 Institute for Biochemistry and Biology, University of Potsdam, 14476, Potsdam, Germany; 3 Sainsbury Laboratory, Cambridge University, Cambridge, CB2 1LR, United Kingdom; 4 Graduate School of Biological Sciences, Nara Institute of Science and Technology, Ikoma, 630–0192, Japan; 5 Department of Cell and Developmental Biology, John Innes Centre, Norwich, NR4 7UH, United Kingdom; Instituto de Biología Molecular y Celular de Plantas, SPAIN

## Abstract

Mitogen-activated dual-specificity MAPK phosphatases are important negative regulators in the MAPK signalling pathways responsible for many essential processes in plants. In a screen for mutants with reduced organ size we have identified a mutation in the active site of the dual-specificity MAPK phosphatase INDOLE-3-BUTYRIC ACID-RESPONSE5 (IBR5) that we named *tinkerbell* (*tink*) due to its small size. Analysis of the *tink* mutant indicates that IBR5 acts as a novel regulator of organ size that changes the rate of growth in petals and leaves. Organ size and shape regulation by IBR5 acts independently of the *KLU* growth-regulatory pathway. Microarray analysis of *tink*/*ibr5-6* mutants identified a likely role for this phosphatase in male gametophyte development. We show that IBR5 may influence the size and shape of petals through auxin and TCP growth regulatory pathways.

## Introduction

Leaf and floral organs grow by two basic cellular processes, cell proliferation and cell expansion to reach a given organ size [1]. Characterization of mutants with defects in proliferation and/or expansion has provided insight into how growth is regulated, with many of these regulatory factors appearing to act in independent pathways and having diverse predicted molecular functions [2]. A considerable number of genes have been identified that influence organ growth. These include genes involved in hormone signalling pathways, regulators of the timing and rate of proliferative or expansive growth and genes controlling identity and patterning of organs [3].

A number of auxin responsive genes have been identified in organ growth control. The auxin-induced *ARGOS* (*AUXIN-REGULATED GENE INVOLVED IN ORGAN SIZE*) gene contributes to regulating the timing of proliferation arrest [4]. *ARGOS* encodes a novel, plant specific protein which acts upstream of *AINTEGUMENTA* (*ANT*), encoding a member of the AP2/ERF transcription factor family. *ARGOS* promotes growth by stimulating *ANT* expression; *ANT* activity maintains the proliferative potential of cells in leaves and floral organs, with loss or gain of function leading to reduced or increased lateral organs, respectively [4–6]. Mitogen-activated protein kinases (MAPKs) have been implicated in auxin signalling and studies show a rapid MAPK activation in response to auxin in Arabidopsis seedling roots [7]. A screen for resistance to the inhibitory effects of the auxin precursor indole-3-butyric acid (IBA) on root growth identified a mutation in a MAPK phosphatase, *INDOLE-3-BUTYRIC ACID-RESPONSE5* (*IBR5*) [8]. *IBR5* is proposed to act as a positive regulator of auxin signalling in roots by inactivating MAPKs, including MPK12 [9]. MAPKs constitute a highly conserved family of enzymes in eukaryotes, and in plants MAPK-based signal transduction modules regulate a large number of physiological processes, including responses to environmental stresses and phytohormones [10].

Activation of MAPKs is regulated via dual phosphorylation of the conserved TXY motif located in the activation loop by upstream kinases (MAPKKs), and this activation can be reversed by dephosphorylation through protein phosphatases, including specialized dual-specificity MAPK phosphatases (MKPs) [11, 12]. There are five putative MKPs in Arabidopsis (AtMKP1, AtMKP2, DsPTP1, PHS1 and IBR5) which have the complete dual-specificty phosphatase active-site motif VxVHCx2GxSRSx5AYLM [8, 13–17]. Dephosyphorylation activitiy against Arabidopsis MAPKs has been shown for AtMKP2 (targeting MPK3 and MPK6), DsPTP1 (targeting MPK4) and IBR5 (targeting MPK12) *in vitro* [14, 15, 18]. IBR5 was confirmed to interact with MPK12 *in vivo*, and MPK12 acts as a negative regulator of auxin signalling [9]. It remains unclear how IBR5 modulates auxin responses in plants as it does not act through TIR1 or through destabilizing Aux/IAA repressor proteins [19]. Sensitivity to ABA also appears to act partially independently of auxin responses in *ibr5* mutants as a suppressor that carries a mutation in PDR9/ABCG36, could restore *ibr5* responses to a subset of auxins, but not to ABA [20].

Although the root phenotypes of *ibr5* mutant plants have been described, above ground growth phenotypes have not been explored in detail. Mutant *ibr5* plants have been described as having a shorter stature, epinastic leaves and defects in vascular patterning. A role for IBR5 in organ size control has not previously been characterized and in this paper we investigate which pathways are disrupted. A *tinkerbell* (*tink/ibr5-6*) mutant was identified in an EMS mutagenesis screen to identify novel regulators of growth control. *tink*/*ibr5-6* plants have reduced petal size and a smaller stature compared to wild-type plants. Mapping revealed that the *tink1* mutation disrupts the dual-specificity phosphatase active-site of IBR5. The growth dynamics of *tink*/*ibr5-6* identified a role in regulating the rate of proliferative growth to maintain correct size and shape of organs. Microarray analysis suggests IBR5 plays a role in male gametophyte development, auxin and TCP growth regulatory pathways.

## Results

### The *tink* mutation affects organ size and shape by altering the rate of proliferative growth

**Fig 1:**
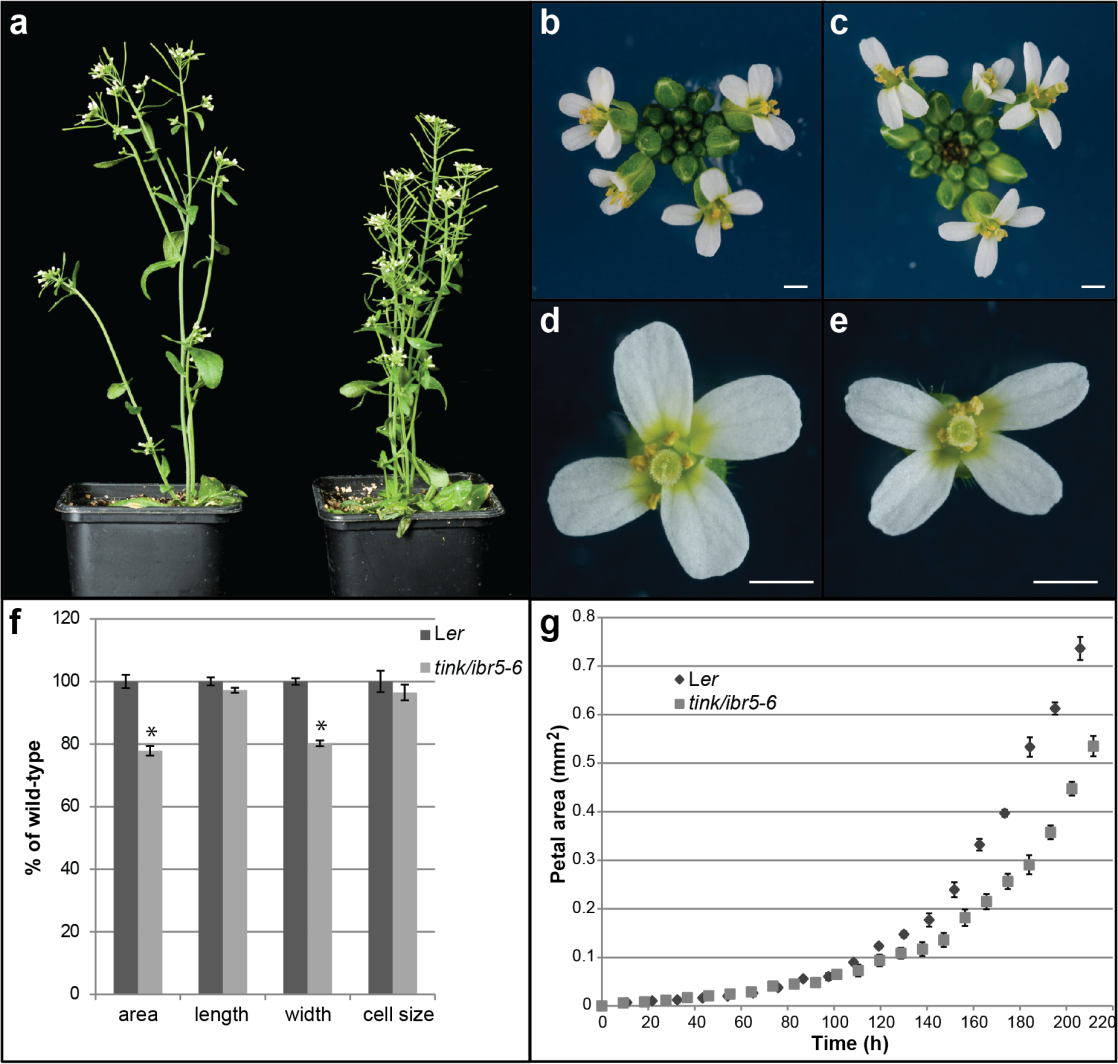
Above ground phenotypes of *tink/ibr5-6* mutants. a. Mutants in *tink1/ibr5-6* (right) display reduced plant height and bushier phenotypes compared to L*er* plants (left). An increased number of flowers in the inflorescence (c) and narrow petals (e) are observed in *tink1/ibr5-6* compared to L*er* (b, d) flowers. f. Measurement of *tink1/ibr5-6* and L*er* petal size showing statistically significant (shown by *) reduction in petal area (p value ≤ 4.5e-14) and petal width (p value ≤ 2e-37) in two tailed t-tests assuming unequal variance. g. Kinematic analysis of *tink1/ibr5-6* and L*er* petal size during development. Scale bar is 1 mm. Values are shown as mean ± SEM where n = 20.

EMS mutagenesis of the *klu-2* mutant in the *Arabidopsis thaliana* Landsberg *erecta* background was undertaken and approximately 2500 individual M2 lines were screened for reduced plant height and reduced petal size. Petals were used for screening as growth of floral organs is more consistent than leaf growth [21]. A mutant line that displayed significantly reduced petal and leaf size compared to *klu-2* single mutants was selected for further investigation and backcrossed three times to wild type to separate the novel mutation and the *klu-2* mutation. The novel mutation was named *tinkerbell* (*tink*) for its small stature (Fig 1A–1E and S1A Fig). Homozygous *tink* single-mutant plants show a 20% reduction in petal size compared to wild-type and this was due to a reduction in petal width (Fig 1F). Size of petals in heterozygous *TINK/tink* plants was indistinguishable from wild-type indicating this is a recessive mutation. To investigate the cause of the reduced petal size we measured cell size in mature petals of *tink* and wild-type plants. Cell size is not altered in *tink* plants compared to wild-type indicating that the reduced size of *tink* petals results from fewer cells (Fig 1F).

To determine how TINK regulates organ size, we followed the growth dynamics of petal and leaf primordia in *tink* mutant and wild-type plants (Fig 1 and S1 Fig). Similar to petals, rosette area was significantly reduced from day 8 in *tink* plants compared to wild-type (illustrated by thick red line in S1B Fig). Mutant *tink* plants were shown to have a slightly decreased plastochron compared to wild-type which was visible by the increased number of flowers in the *tink* inflorescence (Fig 1C). Kinematic analysis of petal and leaf growth shows that *tink* plants have a reduced rate of organ growth compared to wild-type (Fig 1G and S1B Fig). This is particularly interesting as previous studies suggest that most regulators of organ size affect the transition between cell division and expansion rather than the rate of cell division itself [3].

### The dual-specificity phosphatase indole-3-butyric acid-reponse5 (IBR5) is mutated in *tink* mutants

**Fig 2:**
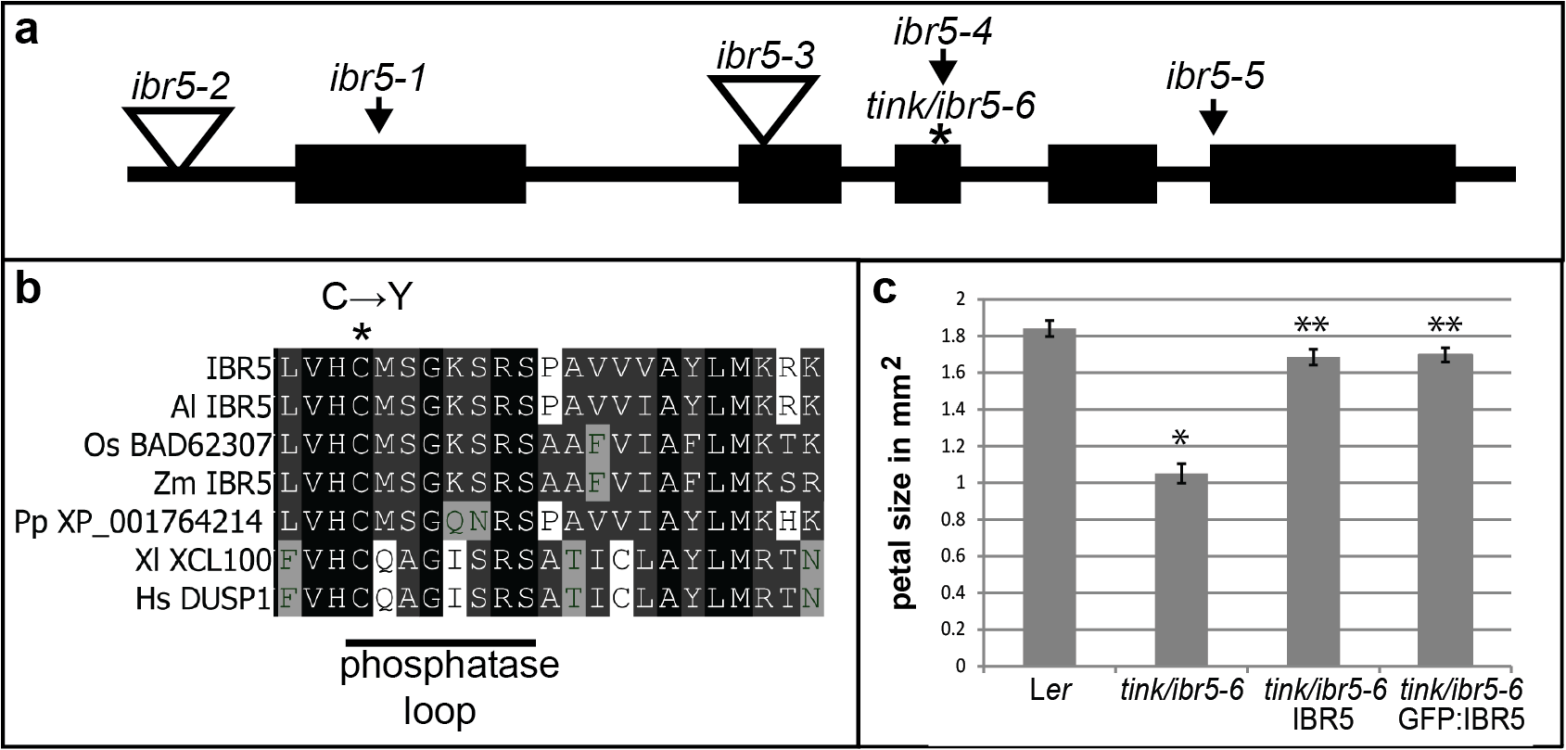
Identification of the *tink/ibr5-6* causal mutation. a. Schematic representation of the *IBR5* gene showing point mutation sites of mutants generated by EMS (indicated by arrows), *ibr5-1* (causing a premature stop codon), *ibr5-4* (G^727^ to A mutation in the third exon that changes G^132^ to E), *ibr5-5* (G to A mutation in the intron of the last intron-exon junction) and *tink/ibr5-6* in the third exon (asterix, G^721^ to A transition in *tink*/*ibr5-6* changes C^129^ to Y in the conserved dual-specificity phosphatase catalytic domain), and T-DNA insertion sites of *ibr5-2* and *ibr5-3* in the promoter and second exon (open triangles). b. Alignment of IBR5 with plant homologues from *Arabidopsis lyrata* (Al), rice (Os), maize (Zm), *Physcomitrella patens* (Pp) and animal homologs from *Xenopus laevis* (Xl) and humans (Hs) showing the conserved phosphatase loop region. Identical residues are shaded in black and similar residues in at least two and four sequences are shaded in light and dark grey respectively. The G to A transition in *tink/ibr5-6* that changes the active-site Cysteine residue to a Tyrosine is indicated with an asterisk. c. Petal size measurements of L*er*, *tink/ibr5-6*, and *tink/ibr5-6* plants complemented with *p35S*::*GFP*:*IBR5* or *p35S*::*IBR5* constructs. The significant reduction in size of *tink/ibr5-6* petals compared to L*er* (shown by *, p value ≤ 2.6e-16, two tailed t-test) is partially rescued in *tink1/ibr5-6* GFP:IBR5 and *tink1/ibr5-6* IBR5 petals. Petal size of *tink1/ibr5-6* GFP:IBR5 and *tink1/ibr5-6* IBR5 is significantly larger than that of *tink/ibr5-6* (shown by **; p value ≤ 6e-12, IBR5:GFP and p ≤ 1.3e-11, IBR5) in two tailed t-tests assuming unequal variance. Values are shown as mean ± SEM, with n = 20.

An F2 population of a backcross of *tink* mutants to Col-0 wild type was used for whole genome sequencing to identify the causal mutation. Rough mapping and analysis of SNPs distribution (S1C Fig) indicated that the mutation was located on the short arm of Chromosome 2. Further analysis of SNPs within this region revealed a G-to-A transition typical of EMS mutagenesis within the coding region of *At2g04550* that was associated with the *tink* mutant (Fig 2A). *At2g04550* corresponds to the previously characterised *IBR5* gene that encodes a dual specificity protein phosphatase 1E [8]. Therefore *tink* represents a new mutant allele of IBR5 that will also be referred to as *ibr5-6*. Dual specificity protein phosphatases are characterized by a highly conserved active site motif VxVHCx2GxSRSx5AYLM, with the cysteine and arginine residues participating with the conserved aspartate in catalysis [18, 22]. The cysteine of this signature begins the dephosphorylation process with a nucleophilic attack on the phosphorus atom of the phosphotyrosine or phosphothreonine substrate. Disruption of this conserved cysteine has been shown to result in catalytic inactivity [19]. The G-to-A transition in *tink*/*ibr5-6* changes the active cysteine residue to a tyrosine (Fig 2B). Complementation of the *tink*/*ibr5-6* mutant with both *p35S*::*IBR5* and *p35S*::*GFP*:*IBR5* construct partially recovered wild-type petal size, indicating that loss of phosphatase activity of IBR5 contributes to the *tink*/*ibr5-6* phenotype (Fig 2C).

**Fig 3:**
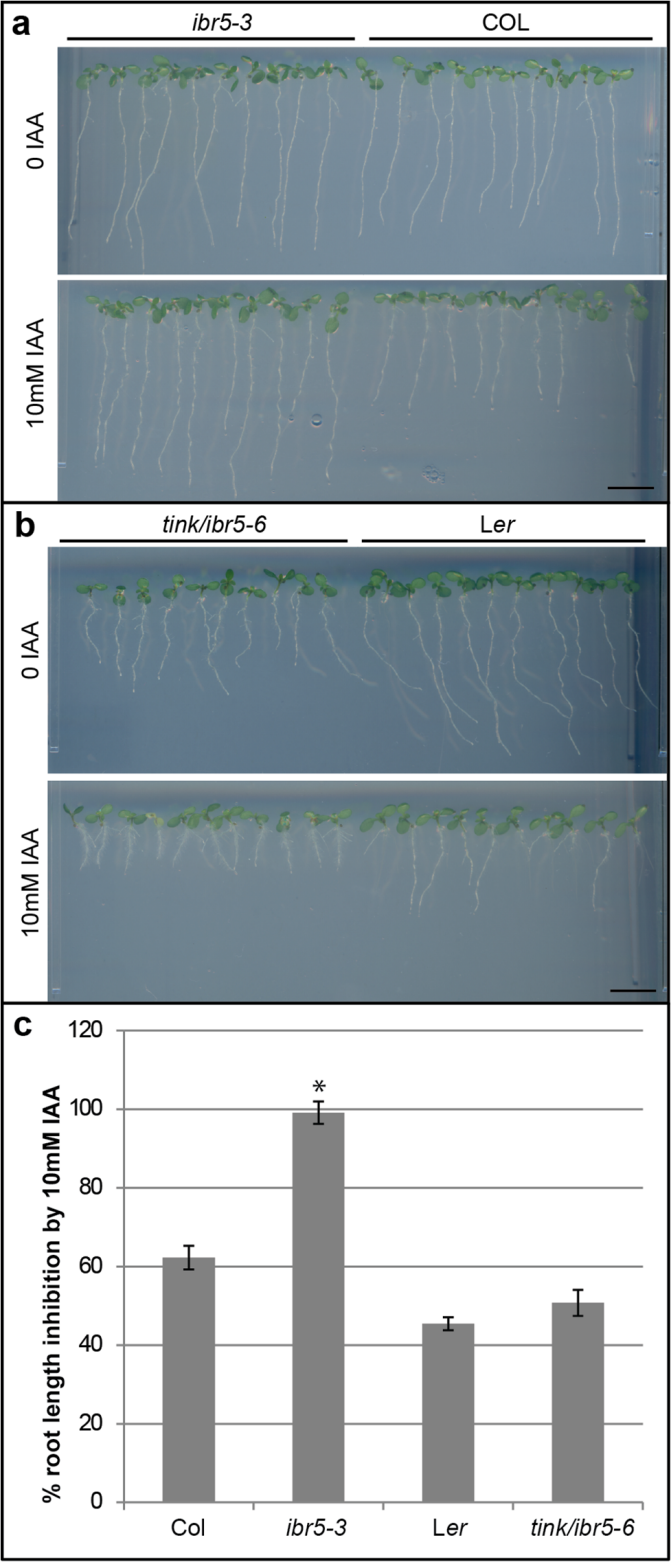
Root phenotype of *ibr5* alleles compared to wild-type. a. On standard growth medium (top panel) the *ibr5-3* allele is indistinguishable from the wild-type (Col) whereas in medium containing 10 mM IAA, the *ibr5-3* allele is insensitive to the inhibition of root growth seen in the wild-type (bottom panel). b. The *tink/ibr5-6* allele shows reduced root growth compared to L*er* on standard growth medium (upper panel) and medium containing 10 mM IAA (bottom panel). c. Inhibition of root length of Col, *ibr5-3*, L*er* and *tink/ibr5-6* plants grown on 10 mM IAA compared to un-supplemented medium. Col plants show a 38% reduction in root growth, compared to *ibr5-3* mutants which are insensitive to the root growth inhibition (shown by *, p value ≤ 5.7e-14). L*er* roots show a 55**%** decrease in root length when grown on 10 mM IAA compared to un-supplemented medium and *tink/ibr5-6* plants show a similar root inhibition phenotype (p value ≤ 0.3). Scale is 1 cm. Values are shown as mean ± SEM, with n = 20.

To investigate if *tink*/*ibr5-6* shows the characteristic reduced auxin sensitivity of other *ibr5* mutants we performed root growth assays with or without the auxin IAA (Fig 3). Unlike *ibr5-3*, *tink*/*ibr5-6* shows a very weak auxin insensitivity phenotype in the presence of 100 nm IAA (Fig 3B). The *tink*/*ibr5-6* mutants show reduced root growth on standard growth media and a slight inhibition of root growth in the presence of auxin compared to the wild-type (Fig 3B and 3C). Previous complementation studies of IBR5 with a C-to-S phosphatase mutation in the *ibr5-1* mutant background and the *ibr5-4* allele which has a G-E transition in the catalytic domain show a weaker response than full loss of function *ibr5* alleles [19, 23]. Additionally, *ibr5-1* IBR5^C-S^ lines fail to fully rescue 2,4-D resistance of *ibr5-1* alleles [19]. These results suggest loss of IBR5 phosphatase activity has pleiotropic effects on root growth.

Investigation of *ibr5-3* mutant flowers revealed a narrow petal phenotype and *tink*/*ibr5-6* displays vascular defects in petals similar to that observed in *ibr5-3* plants (S1D–S1F Fig; [19]). A reduced size of adult *ibr5* mutant plants compared to wild-type has previously been observed [8,19] and here we show that both petals and leaves are significantly smaller than wild-type. The above ground phenotypes of *ibr5* mutant alleles varies. Presumed loss of function *alleles ibr5-1* and *ibr5-3*, as well as an allele with altered transcript length, *ibr5-5* show reduced plant height [23]. Alleles with altered IBR5 catalytic activity, *ibr5-4* and *ibr5-1* IBR5^C-S^ do not affect plant height whereas the *tink/ibr5-6* allele presented here appears to be consistent with the loss of function alleles.

Previous studies of the *IBR5* promoter using GUS reporters showed *IBR5* is expressed in roots, leaves and flowers and did not reveal any GUS expression in mature petals [8]. Other resources such as AtGenExpress microarray data suggest *IBR5* is expressed in petals of young flowers (http://www.weigelworld.org/resources/microarray/AtGenExpress; [24]. It is possible *pIBR5*:GUS was not detected in petals in previous studies due to regulatory elements being missed in the promoter fragment (-2005 to -30) used, rather than the 3850bp upstream to the next upstream gene. Alternatively, *IBR5* could be expressed earlier in petal development than the mature flower investigated or *IBR5* acts non-cell autonomously to affect petal size. Identification of different splice variants of *IBR5* that have some distinct functions may also play a role in post-transcriptional regulation of *IBR5* in the peta ([23]). Previous studies show *p35S*:YFP:IBR5 fusion proteins located to the nucleus in root epidermal cells [9, 23]. Similarly our studies of the complementing *p35S*::*GFP*:*IBR5* construct also show nuclear localization in the petal (S2 Fig).

### Organ size and shape regulation by TINK/IBR5 acts independently of KLU regulatory pathways

**Fig 4:**
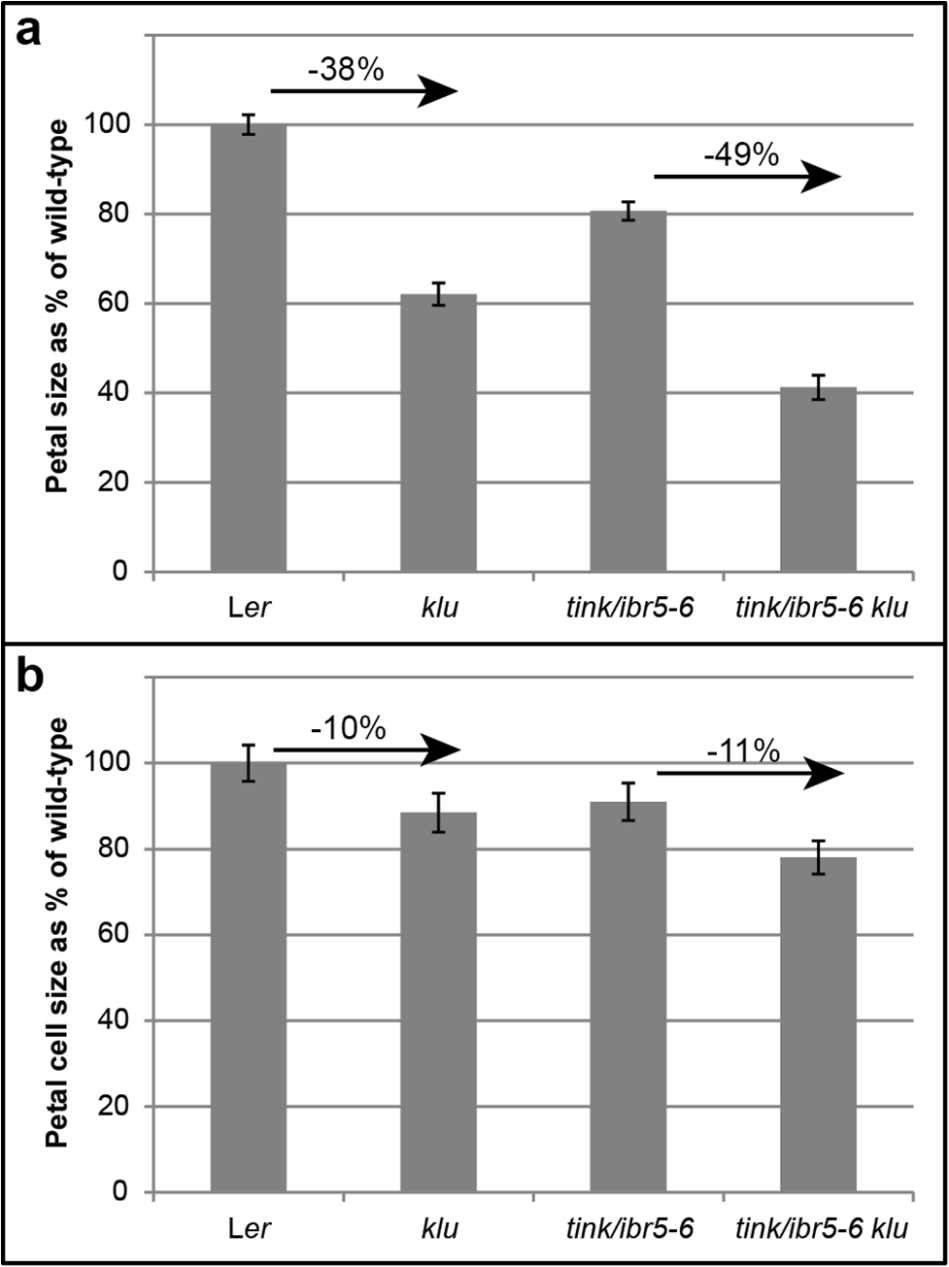
Double mutant analysis of *tink/ibr5-6 klu-2* mutants compared to single mutants and wild-type. a. A significant reduction in petal area (p value ≤ 2.4e-7) is seen in *klu-2* mutants compared to wild-type and the same relative decrease in petal size occurs in double mutants of *tink/ibr5-6* with *klu-2*. b. Cell size in *klu-2* mutants is significantly reduced (p value ≤ 0.02) compared to L*er* and the same relative decrease in cell size occurs in *tink/ibr5-6 klu-2* mutants. Values are shown as mean ± SEM, with n = 10.

The *tink/ibr5-6* mutant was identified in a mutagenesis screen as an enhancer of the *klu-2* mutant phenotype. The cytochrome P450 KLUH (KLU)/CYP78A5 is presumed to generate a growth-promoting signal that acts in a regulatory mechanism to coordinate the growth of individual organs [25]. Increased activity of *KLU* causes organ overgrowth, while *klu* mutants form smaller aerial organs consisting of fewer cells. Detailed investigation of double mutant *tink/ibr5-6 klu-2* plants shows an additive effect to decrease petal size (Fig 4). This suggests that *IBR5* acts independently of *KLU* regulatory pathways.

### Microarray analysis of *tink/ibr5-6* mutants identifies a role for IBR5 in male gametophyte development

To gain further insight into the function of IBR5, transcriptional profiling of *tink*/*ibr5-6* closed flowers was undertaken. A vast number of genes are mis-regulated in this mutant with an absolute log2 fold change above 1 compared to the wild-type (S1 Table). Gene ontology (GO) analysis, which categorises genes based of the gene product properties, such as cellular component, molecular function and biological process, shows an over-representation of genes involved in reproduction and more specifically male gametophyte development (Table 1). Expression of a subset of genes predicted to be involved in pollen development were confirmed as mis-regulated in the *tink*/*ibr5-6* mutant using quantitative real-time (Q)-PCR (S3 Fig). To test for a defect in pollen function, pollen morphology of *tink*/*ibr5-6* was observed, and the transmission efficiency of *tink*/*ibr5-6* pollen was investigated by crossing *tink/+* heterozygotes as male to wild-type female parents. Anthers and pollen of *tink*/*ibr5-6* mutants did not show any gross morphological defects compared to wild-type. Also, no significant difference in transmission efficiency through the *tink*/*ibr5-6* male gametophyte is evident (S3 Fig). Thus, despite the expression signature in *tink*/*ibr5-6* mutant flowers, mutant pollen does not seem to be impaired in its function under standard laboratory conditions, and more specific conditions may be required to reveal a pollen phenotype.

**Table 1:** List of GO terms enriched in *tink/ibr5-6* vs L*er* microarray dataset.

GOID	GOterm	Annotated	Significant	Expected	Fisher.p.value
GO:0090406	pollen tube	39	20	1.74	2.46E-17
GO:0042995	cell projection	40	20	1.79	4.72E-17
GO:0048610	cellular process involved in reproduction	164	31	8.53	3.48E-10
GO:0009860	pollen tube growth	79	21	4.11	3.71E-10
GO:0035295	tube development	102	23	5.31	1.88E-09
GO:0048868	pollen tube development	102	23	5.31	1.88E-09
GO:0004857	enzyme inhibitor activity	138	26	7.29	1.35E-08
GO:0009932	cell tip growth	99	21	5.15	2.98E-08
GO:0009856	pollination	158	27	8.22	4.44E-08
GO:0046910	pectinesterase inhibitor activity	43	13	2.27	1.93E-07
GO:0031225	anchored to membrane	219	28	9.79	5.49E-07
GO:0030234	enzyme regulator activity	297	37	15.69	1.15E-06
GO:0016798	hydrolase activity, acting on glycosyl bonds	396	44	20.92	2.69E-06
GO:0032989	cellular component morphogenesis	271	33	14.1	5.03E-06
GO:0030154	cell differentiation	350	39	18.2	6.36E-06
GO:0048869	cellular developmental process	489	49	25.43	8.76E-06
GO:0022414	reproductive process	1012	84	52.64	1.40E-05
GO:0000003	reproduction	1030	84	53.57	2.66E-05
GO:0032501	multicellular organismal process	1867	135	97.11	4.41E-05
GO:0048235	pollen sperm cell differentiation	27	8	1.4	4.77E-05
GO:0031224	intrinsic to membrane	699	53	31.26	1.20E-04
GO:0009653	anatomical structure morphogenesis	559	50	29.07	1.36E-04
GO:0004091	carboxylesterase activity	290	31	15.32	1.58E-04
GO:0048232	male gamete generation	34	8	1.77	2.82E-04
GO:0055046	microgametogenesis	38	8	1.98	6.31E-04
GO:0007276	gamete generation	40	8	2.08	9.05E-04
GO:0005623	cell	11775	556	526.61	7.00E-03
GO:0044464	cell part	11775	556	526.61	7.00E-03
GO:0016787	hydrolase activity	2578	163	136.22	7.44E-03
GO:0019953	sexual reproduction	80	10	4.16	8.38E-03
GO:0048609	multicellular organismal reproductive process	88	9	4.58	3.91E-02

### IBR5 likely regulates growth through auxin and TCP growth regulatory pathways

**Fig 5:**
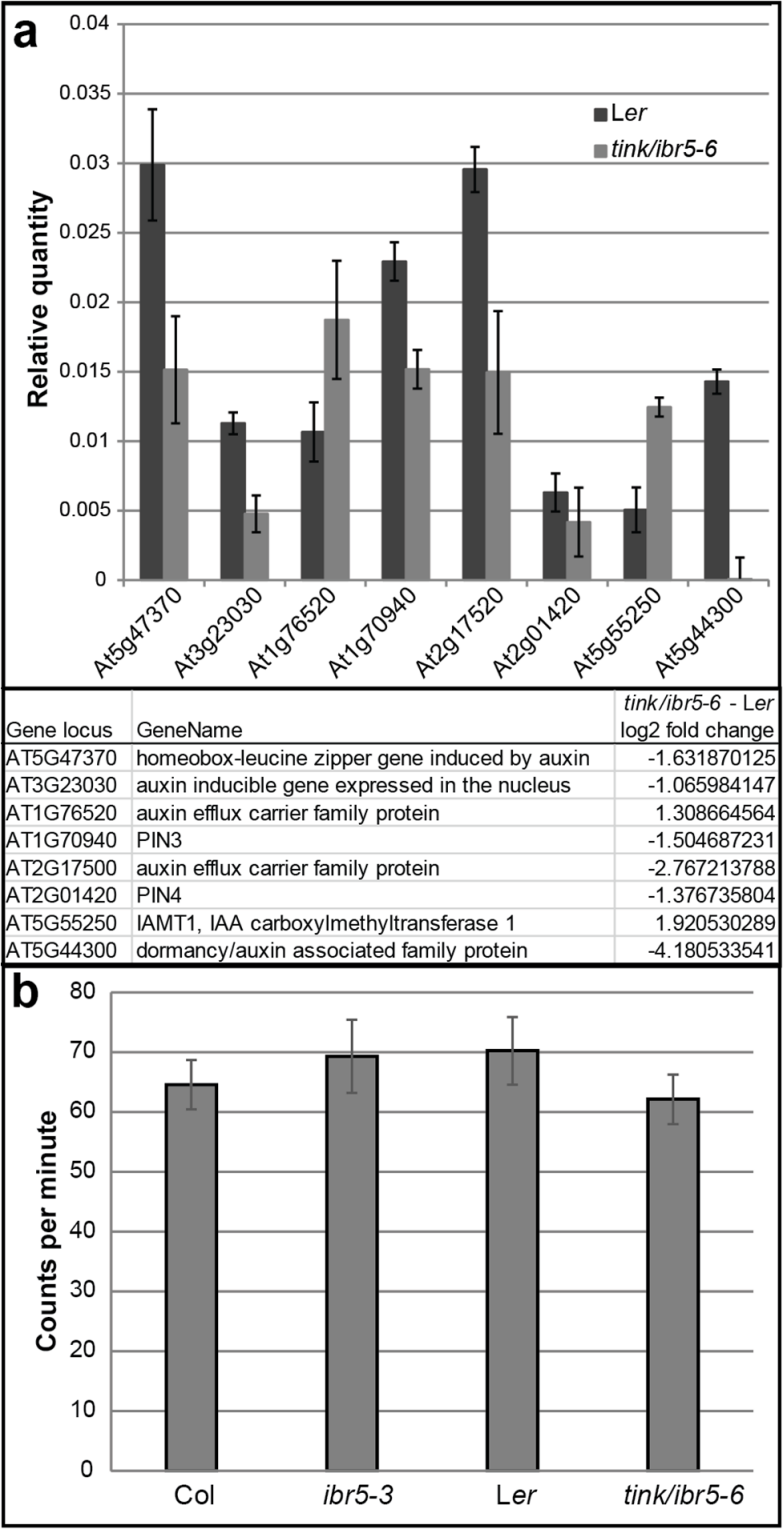
Investigation of auxin pathways in *tink/ibr5-6* mutants. a. Real time quantitative (Q)-PCR validation (upper panel) of genes implicated in auxin biogenesis or transport identified in microarray analysis (lower panel) as being altered in *tink/ibr5-6* mutants compared to wild-type flowers. Values in Q-PCR analysis are shown as mean ± SEM with expression levels normalized to that of the *TUB6* gene for 3 biological and 3 technical replicates. b. Quantitation of bulk polar IAA transport in stem segments of the indicated genotypes. Values are represented as mean ± SEM of radiolabel transported (in cpm). There is no significant difference between wild-type and the respective *ibr5* mutant alleles. At least 18 stem segments were assayed per genotype.

Given the previous association of IBR5 with regulation of auxin pathways and results showing reduced expression of *pDR5*:GUS in the *ibr5-1* mutant [8], we were interested to know if auxin responsive genes are mis-regulated in *tink*/*ibr5-6*. A set of genes identified in microarray studies as being significantly up- or down-regulated in *tink*/*ibr5-6* compared to wild-type were validated by Q-PCR. These genes are annotated as being involved in auxin synthesis, transport, regulation and responses (S2 Table, Fig 5A). Intriguingly this list included a number of genes involved in auxin efflux (PINs). This supports previous studies that IBR5 is involved in auxin signalling pathways. Auxin transport assays performed on basal stem segments of *ibr5-3* and *tink/ibr5-6* mutants compared to the respective wild-types showed no significant differences (Fig 5B). To determine if changes in auxin distribution are responsible for the altered petal phenotype, the *pDR5*:GFP reporter was introduced into *tink*/*ibr5-6 and ibr5-3* mutants [9]. In wild-type petals *pDR5*:GFP is localized at the tip of developing petals and in the vasculature [26]. No difference to wild-type *pDR5*:GFP pattern of expression was observed in the *ibr5* mutant lines (S4 Fig). A reduction in the level of *pDR5*:GUS in roots and leaves has previously been observed in *ibr5-1* mutants, even in tissues where *pIBR5*:GUS was not observed [8, 19]. From our investigation of *pDR5*:GFP expression in petals of *ibr5* mutants, either no change in auxin levels occurs or our methods are not sensitive enough to detect potentially subtle changes in *pDR5*:GFP expression.

As cell proliferation is altered in *tink/ibr5-6* mutants, the microarray data was searched for mis-regulated cyclin and related genes. A cyclin-dependent protein kinase, CYCP3;1 (AT2G45080), is significantly upregulated and a number of F-box family proteins altered in the *tink/ibr5-6* mutants compared to wild-type (S3 Table). To identify other pathways through which IBR5 could be acting, MASTA analysis was used to compare the *tink*/*ibr5-6* transcriptional profile to other available microarray data. This analysis identified a large overlap with the transcriptional profile of *tcp14* and *tcp15* mutants (S4 Table and S5 Table, S5 Fig). TCP transcription factors constitute a small family of plant-specific bHLH-containing, DNA-binding proteins that have been implicated in the control of cell proliferation in plants. To investigate if IBR5 overlaps with other TCP pathways, the *tink/ibr5-6* dataset was also compared to a *jaw-D* microarray (S5 Fig). The JAW microRNA targets a number of class II TCPs, in particular *TCP3* and *TCP4*, and dominant *jaw-D* alleles result in plants with uneven leaf shape and curvature [27]. Unlike *tcp14 tcp15*, no obvious overlap with *tink/ibr5-6* and *jaw-D* microarray datasets occurs (S5 Fig). Reminiscent of the *tink*/*ibr5-6* mutant, *tcp14* and *tcp15* mutants have a reduced plant height phenotype that results from a reduction in internode length. Leaf shape is also subtly altered in *tcp14 tcp15* mutants indicating a role in regulating organ size and shape via changes in cell proliferation [28]. TCP15 is also implicated in regulating auxin homeostasis as the expression of an auxin-responsive promoter is induced by TCP15-EAR [29]. Further investigation to determine if IBR5 and TCP14 and TCP15 act in the same pathway to regulate organ size is an avenue for future research.

## Discussion

### *tink/ibr5-6* alters the rate of proliferative growth

We have identified a novel role for the dual-specificity protein phosphatase IBR5 in regulating the shape and size of Arabidopsis organs. Loss of *IBR5* function leads to narrow petals and leaves and its effect appears to be due to an altered rate of proliferative growth rather than changes in cell size. The reduced growth rate in *tink*/*ibr5-6* in petals leads to 80% of the wild-type cell number. The plastochron of *tink*/*ibr5-6* mutants is also shorter leading to an increased number of flowers in the inflorescence (Fig 1). The transition between cell proliferation and cell expansion is known to be a critical decision point during primordium growth. Unlike many other regulators of cell proliferative growth, IBR5 does not alter the length of time over which growth occurs and appears to be a novel regulator of growth. Consistent with an effect primarily on the rate of growth, rather than its duration, the *tink/ibr5-6* mutation did not show any interaction with the *klu* mutation, which affects the timing of growth arrest, but not the growth rate.

### Microarray analysis of *tink/ibr5-6* revealed potentially novel roles in male gametogenesis and TCP regulatory pathways

Microarray analysis of *tink*/*ibr5-6* mutant flowers identified a large number of mis-regulated genes compared to wild-type (S1 Table). An obvious pathway for organ size regulation was not identified whereas GO analysis of the *tink*/*ibr5-6* microarray data suggests a significant overrepresentation of genes expressed in male gametophyte development and function. Transmission efficiency of *tink/ibr5-6* gametes is not affected and the mechanism resulting in the change in gene expression remains somewhat elusive. Interestingly, a study of the phosphoproteome of mature Arabidopsis pollen identified an overrepresentation of mitogen-activated protein kinases [30]. The dual phosphorylation of MPK8 and MPK15 was confirmed yet no role in pollen development has been described to date. It is possible IBR5 plays a role in the male gametophyte through modulating activity of these or other MPKs. Although IBR5 did not interact with MPK8 or MPK15 in yeast-2-hybrid studies, an *in vivo* interaction cannot be ruled out [9].

Microarray comparison (MASTA) analysis revealed an interesting overlap of the *tink*/*ibr5-6* profile with that of *tcp14 tcp15* [31]. TCP transcription factors are key regulators of cell proliferation in growing organs and the balance between the growth-promoting class I factors and the negatively acting class II TCPs has been proposed to regulate the arrest of proliferative growth [32, 33]. Class I TCP factors are proposed to stimulate division required to produce the correct number of cells in young lateral primordia followed by a suppression of cell growth and division by class II TCP genes as cells exit the proliferative zone [34]. This view has been challenged in recent times as the class I TCPs, TCP14 and TCP15, can act to either promote or repress cell proliferation depending on the developmental context [28].

TCP14 and TCP15 are closely related class I TCP genes that modulate cell proliferation in the developing leaf blade and floral tissues and promote cell division in young internodes [28]. Overexpression of TCP14 (*pAS1*:TCP14) resulted in inhibition of internode elongation, inhibition of petal growth, reduced fertility and promotion of trichome development on sepals [35]. In *tcp14 tcp15* double mutants and TCP14 overexpression lines the activity of the promoter of the mitotic factor CYCB1;2 is reduced or increased in stems, respectively [28, 35].

It is possible the *ibr5* mutant growth phenotypes are mediated in part through altered activity of TCP14 and TCP15 transcription factors. It is tempting to suggest that this may occur through changes in phosphorylation status of these proteins. Recently a recombinant TCP8 was shown to be phosphorylated at Ser211 when expressed in Hi5 insect cells [36]. Characterisation of TCP14 and TCP15 in the *ibr5* mutant background and generation of triple mutant *tink/ibr5-6 tcp14 tcp15* mutants are interesting avenues for further investigation. Interestingly, TCP15 has been implicated in auxin homeostasis as it has been shown to bind the promoter regions of IAA3/SHY2 and the auxin responsive gene At1g29460 [29]. IBR5 is known to play a role in regulating auxin pathways yet the mechanism has remained cryptic.

### IBR5 likely mediates growth through auxin pathways

The IBR5 loss of function mutant alleles *ibr5-1* and *ibr5-3* show less sensitivity to inhibitory concentrations of auxin in root growth assays. Expression of pDR5:GUS is reduced in these *ibr5* alleles suggesting that IBR5 normally acts to promote auxin responses [8, 19, 20]. The role of IBR5 in auxin pathways remains unclear as *IBR5* transcript levels do not alter in response to auxin, IBR5 acts independently of the TIR1 and AXR1 and AUX1 pathways and Aux/IAA proteins are not stabilized in *ibr5* [19].

MPK12 has been identified as interacting with IBR5 and shown to be a negative regulator of auxin signalling in roots [9]. MPK12 activity is activated by auxin *in vivo* and reduced levels of MPK12 result in hypersensitivity to auxin [9]. The association of MPK12 with IBR5 has reinforced their roles in regulation of auxin signalling pathways. Unlike other *ibr5* alleles, *tink/ibr5-6* does not show strong reduced sensitivity to the inhibitory effects of auxin on root growth. The *ibr5-1* mutation causes a premature stop codon that would result in a truncated product lacking the conserved phosphatase domain and the C-terminal region responsible for binding MAPK12 [8, 9]. As the *tink/ibr5-6* mutation results in a full length protein with likely loss of phosphatase activity it is possible that it is able to sequester MPK12 and dampen its normal activity. This is likely given previous complementation studies of the *ibr5-1* mutant using a mutated IBR5^c129s^ only resulted in partial rescue of *ibr5* defects [19]. Studies suggest that IBR5 may have some phosphatase independent functions and may bind and sequester its substrate(s) [19, 23]. This suggestion would fit with the root phenotypes of *tink/ibr5-6* mutants as they display reduced root growth in the absence of auxin. The *tink/ibr5-6* mutation could potentially cause the IBR5^C-Y^ to act as a dominant negative in roots. This could occur through sequestration of its substrate(s) such as MPK12. Plants with reduced levels of MPK12 show hypersensitivity to auxin; however, they do not show root phenotypes in the absence of auxin. The *tink/ibr5-6* root phenotype on standard media may reflect the IBR5^C-T^ more completely suppressing MPK12 and potentially other MAPK substrates.

MPK12 is not expressed in the petals which may explain why *ibr5* mutant phenotypes are more consistent in this organ. Complementation with a *p35S*::*GFP*:*IBR5* construct largely recovered the *tink*/*ibr5-6* petal size phenotype suggesting that the IBR5 phosphatase is indeed altered in the *tink*/*ibr5-6* mutant (Fig 2). IBR5 could have a different MAPK substrate in petals as well as having both phosphate dependent and phosphate independent functions.

A role for IBR5 in modifying auxin transport could be inferred from the identification of *pleiotropic drug resistance9* (*pdr9*) as a suppressor of *ibr5* [20]. *PDR9* encodes an ATP-binding cassette transporter implicated in cellular efflux of the synthetic auxin 2,4-D. Microarrays of *tink/ibr5-6* identified a number of the auxin efflux *PIN* genes with altered expression profiles. A number of auxin responsive genes have been identified in organ growth control. The auxin-induced *ARGOS* (*AUXIN-REGULATED GENE INVOLVED IN ORGAN SIZE*) gene contributes to regulating the timing of proliferation arrest [4]. *ARGOS* encodes a novel, plant specific protein which acts upstream of *AINTEGUMENTA* (*ANT*), encoding a member of the AP2/ERF transcription factor family. *ARGOS* promotes growth by stimulating *ANT* expression; *ANT* activity maintains the proliferative potential of cells in leaves and floral organs, with loss or gain of function leading to reduced or increased lateral organs, respectively [4–6]. Genetic interaction studies with *ant* could potentially uncover an interaction of IBR5 and ANT pathways although given *ANT* and *ANT-like* genes act to regulate the length of proliferative growth, it seems likely that IBR5 acts independently of this pathway.

Auxin transport assays in *ibr5-3* and *tink/ibr5-6* stems did not reveal altered dynamics and localization of a pDR5:GFP reporter did not show any difference in levels or pattern of expression in *ibr5-3* or *tink/ibr5-6* mutant petals. It is possible that subtle changes in auxin signalling at the cellular level are responsible for the *ibr5* above ground phenotypes and examination of PIN protein localization in *ibr5* mutants would be an interesting avenue to further investigate these effects. Alteration of auxin patterning would explain the narrow petal phenotype as auxin is thought to act as a negative polarity organizer [26].

In this paper we have described a new allele of *ibr5* and detail the above ground floral phenotypes. The pathways via which IBR5 regulates growth through auxin and TCP pathways open up further areas of research for this regulator of signalling pathways.

## Experimental Procedures

### Plant Material and growth conditions

The *tink/ibr5-6* mutant was isolated from an EMS-mutagenesized population in the *klu-2* background and backcrossed three times to L*er* plants to isolate *tink/ibr5-6* single mutants. *klu-2* mutants are described in Anastisou et al., (2007). Plant growth conditions were as described in [37].

#### Identification of the TINK causal mutation

The *tink/ibr5-6* mutation was rough mapped in a *tink/ibr5-6* x Col F2 population by using described markers (http://carnegiedpb.stanford.edu/publications/methods/pps.uppl.html). Nuclei from 250 *tink/ibr5-6* x Col F2 plants showing the mutant phenotype were extracted using the CellLytic PN kit semi-pure method (Sigma) to identify SNPs using whole genome sequencing. Library preparation, sequencing and analysis of SNP data are outlined in [38]. Genotyping of the *tink/ibr5-6* causal mutation in At2g04550 was performed using KASP genotyping (LGCgenomics) with primers At2g04550_SNP12_FOR, 5’ GGCACGTGTTCTTGTGCATT 3’, At2g04550_SNP12_fam, 5’ GAAGGTGACCAAGTTCATGCTCTCACCTACTTTTCCCAGACATGC 3’, At2g04550_SNP12_VIC, 5’ GAAGGTCGGAGTCAACGGATTCTCACCTACTTTTCCCAGACATGT 3’.

### Phenotypic Analysis

Organ and cell sizes, as well as growth dynamics of petals, were measured as described by Disch et al., (2006). Values are represented as mean ± SEM throughout. Each value corresponds to at least twenty petals from at least ten plants. Petals were imaged either on a Zeiss SteREO Lumar dissecting microscope or a Leica DM 6000 compound microscope and petal measurements were taken from images using Fiji (http://fiji.sc). For root elongation inhibition assays plants were grown aseptically on half MS media (Sigma) with 0.5%[w/v] sucrose solidified with 0.8% (w/v) agar, either alone or supplemented with 100nm IAA. Plants were grown for 10d at 22°C under standard long day conditions and the length of the primary root was measured. A minimum of 20 roots were measured for each genotype and values are represented as mean ± SEM throughout.

### Generation of GFP-IBR5 constructs

The coding sequence corresponding to *IBR5* was amplified from Arabidopsis leaf cDNA (L*er* ecotype), using Phusion high fidelity DNA polymerase (New England Biolabs) and primers, At2g04550_Xba_Spe_F, 5’ TCTAGACAAACTAGTATGAGGAAGAGAGAAAGAGAG 3’ and At2g04550_R_Sal, 5’ ACGGTCGACCTAAGAGCCATCCATTGCA 3’ according to the manufacturer’s instructions. The *IBR5* sequence was cloned into the FP101 vector using XbaI and Sal restriction sites and GFP inserted into the SpeI site at the N-terminus [39]. This binary vector was transformed into *Agrobacterium tumefaciens* strain GV3101 and transformed into Arabidopsis using the floral dip method [40].

### Microarray Analysis

RNA was extracted from inflorescences with young flower buds from individual *tink/ibr5-6* mutant or L*er* plants with three replicates using the RNEasy kit (Qiagen). Hybridization of Affymetrix ATH1 arrays was performed by NASC Affymetrix service, Nottingham, UK. Arrays were normalized using gcrma with differentially expressed genes identified using the R/Bioconductor packages affy, limma and gcrma. Genes with absolute log2 fold change above 1 and BH corrected p-values below 0.05 were considered differentially expressed. Arrays were also analyzed using R/RankProd to extract the top 200 up- and down-regulated genes for comparison with the MASTA dataset [31]. Normalized signal intensities for *jaw-d* microarrays ([27]; PMID: 12931144) were downloaded from NCBI GEA (accession number GSE518) and analyzed the same way to be added to the comparison. Raw data for our microarrays are available at NCBI GEO under accession number GSE66419.

### Quantitative real-time (Q)-PCR

Q-PCR was used to verify microarray results for a subset of genes involved in male gametogenesis and auxin regulation. RNA was extracted as above and cDNA synthesized from 1μg of RNA using the Superscript III (Invitrogen) reverse transcriptase according to the manufacturer’s instruction. Q-PCR was performed with SYBR-Green PCR Mastermix (Invitrogen) on a Bio-Rad DNA Engine with Chromo4 RT-PCR Detector. Three independent RNA samples for each genotype were assayed in triplicate. Expression levels were normalized to those of the constitutively expressed TUBULIN (At5g12250, [41] gene in each sample and are shown as mean ± SEM. Oligonucleotide sequences are given in S6 Table.

### Auxin transport Assay

Auxin transport assays were performed as described [42] with the following slight modifications. Stem segments (18 mm long) excised from the most basal cauline internodes were incubated for 18 h in 1 × *Arabidopsis* salts medium (ATS) containing 1% sucrose and radiolabeled IAA (1 μM). The amount of transported radiolabel was quantified by scintillation counting (Top CountNXT; Packard Biosciences). Plants were cultivated in the greenhouse under long-day conditions with additional artificial light when needed. Six-week-old plants were used for analysis.

## Supporting Information

S1 Fig Additional phenotypes of *ibr5* mutants and mapping of the *tink/ibr5-6* causal mutation. a. Representative L*er* (left) and *tink/ibr5-6* mutant (right) plants show the rosette leaves of *tink/ibr5-6* mutants are narrower than wild-type. b. Kinematic analysis of *tink1/ibr5-6* and L*er* rosette size during development show *tink1/ibr5-6* has significantly smaller rosette area (shown by red bar, P-values below 0.05 using Wilcoxon Rank Sum tests) c. SNP distribution on chromosome 2 in a mapping population of *tink/ibr5-6* (L*er*) crossed to Columbia. The ratio of homozygous SNPs to heterozygous SNPs was plotted (y-axis). d. Measurement of *ibr5-3* and Col petal size shows a statistically significant (shown by *) reduction in petal area (p value ≤ 3e-7) and petal width (p value ≤ 1e-14) in *ibr5-3* mutants using two tailed t-tests assuming unequal variance. Petals of L*er* (e) and *tink/ibr5-6* (f) mutants show defects in vein patterning in *tink/ibr5-6* compared to wild-type. Scale bar is 1 cm in (a) and 0.1 mm in (e,f).
(TIF)

S2 Fig Localisation of a GFP-IBR5 fusion protein in petal cells of *p35S*::*GFP*:*IBR5* plants. GFP is located in the nucleus, DIC (a), GFP fluorescence signal (b), and merge (c). Scale bar is 10μm.
(TIF)

S3 Fig Investigation of male gametophyte development in *tink/ibr5-6* plants. Phenotype of wild-type (L*er*) (a) and *tink/ibr5-6* (b) anthers show no obvious differences. c. Q-PCR (upper panel) and microarray (lower panel) analysis of genes specifically expressed in male gametophyte development, with altered expression in *tink/ibr5-6* mutants compared to wild-type (L*er*). d. Transmission efficiency of the *tink/ibr5-6* mutation through male gametes. Scale in (a) and (b) is 0.1 mm. Values in (c) are shown as mean ± SEM from 3 biological and 3 technical replicates with expression levels normalized to that of the *TUB6* gene.
(TIF)

S4 Fig Pattern of *pDR5*:*GFP* expression in wild-type (a-c), *ibr5-3* (d-f) and *tink/ibr5-6* (g-i) petals. Bright-field image is shown in a, d and g, GFP fluorescence is shown in b, e and h and merged image is shown in c, f and i. Scale bar is 100 μm.
(TIF)

S5 Fig Venn diagrams showing overlap of *tcp13*, *tcp14* and jaw-D microarray datasets with *tink/ibr5-6* mutants using MASTA analysis. The number of overlapping genes (A, C, E and G) and the inverse overlap (B, D, F, H) significantly up (red) or down (blue) regulated is shown for comparison of *tink/ibr5-6* with *tcp13* (A, B), *tcp14* (C, D), *tcp13/+ tcp14/+* (E, F) and *jaw-D* (G, H).
(TIF)

S1 File Supporting Experimental Procedures and References. (DOCX)

S1 Table De-regulated genes in *tink/ibr5-6 mutant* compared to wildtype. (XLSX)

S2 Table Genes involved in auxin regulation mis-expressed in *tink/ibr5-6* mutants. (XLSX)

S3 Table Cyclin and related genes mis-expressed in *tink/ibr5-6* mutants. (XLSX)

S4 Table MASTA comparative analysis of *tink/ibr5-6* and publically available microarray datasets. (XLSX)

S5 Table Overlapping genes in MASTA comparison of *tink/ibr5-6* and *tcp14* and *tcp15* mutants. (XLSX)

S6 Table Primers used for Q-PCR analysis. (XLSX)
