# Correction: The *Tinkerbell* (*Tink*) Mutation Identifies the Dual-Specificity MAPK Phosphatase INDOLE-3-BUTYRIC ACID-RESPONSE5 (IBR5) as a Novel Regulator of Organ Size in Arabidopsis

**DOI:** 10.1371/journal.pone.0136482

**Published:** 2015-08-20

**Authors:** Kim L. Johnson, Sascha Ramm, Christian Kappel, Sally Ward, Ottoline Leyser, Tomoaki Sakamoto, Tetsuya Kurata, Michael W. Bevan, Michael Lenhard

The IAA concentration in Fig 3 is incorrect and should be listed as 100nM IAA. The authors have provided a corrected version here.

**Fig 3:**
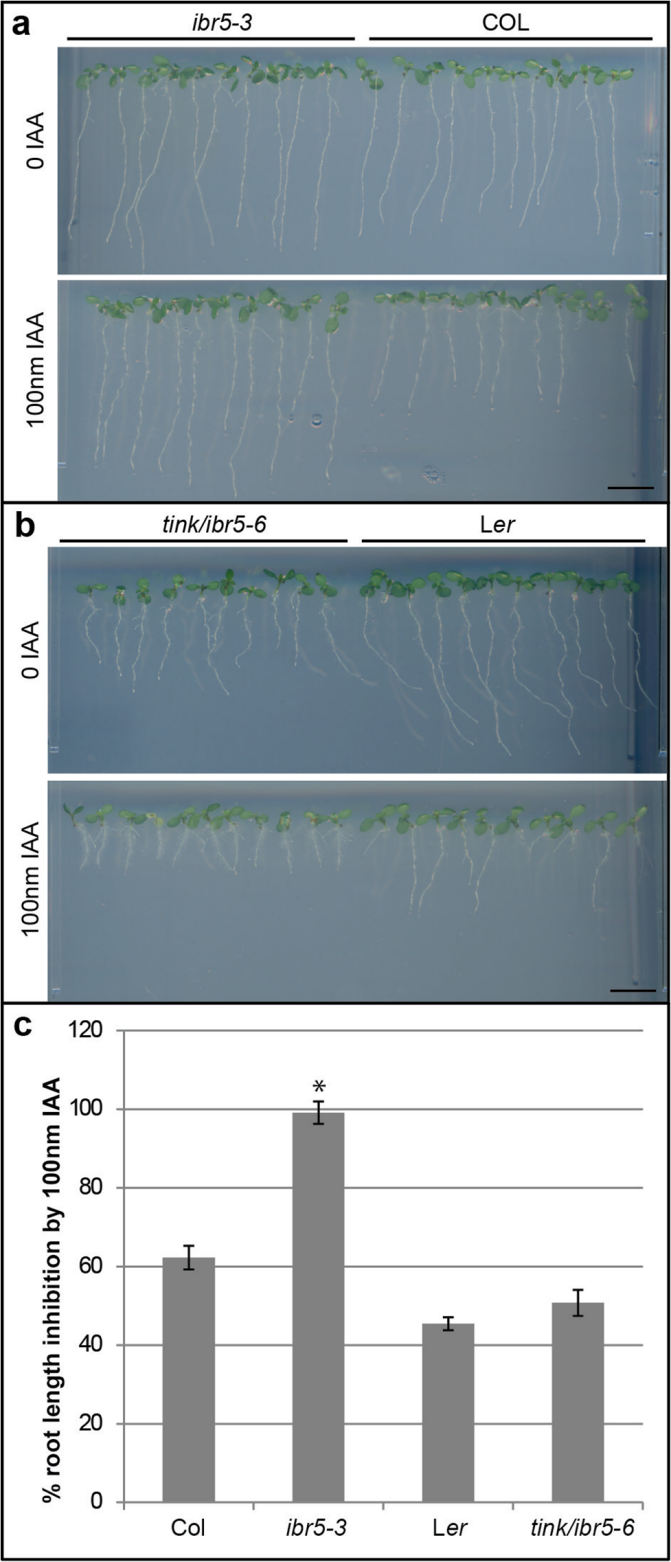
Root phenotype of *ibr5* alleles compared to wild-type. a. On standard growth media (top panel) the *ibr5-3* allele is indistinguishable from the wild-type (Col) whereas in media containing 100nm IAA, the *ibr5-3* allele is insensitive to the inhibition of root growth seen in the wild-type (bottom panel). b. The *tink/ibr5-6* allele shows reduced root growth compared to L*er* on media with or without 100nm IAA and displays a slightly significant difference (p value ≤ 0.3) in root growth inhibition on 100nm IAA compared to the wild-type (c). Scale is 1cm. Values are shown as mean ± SEM where n = 20.
