# Supplementary figures and images for: The Tinkerbell (Tink) Mutation Identifies the Dual-Specificity MAPK Phosphatase INDOLE-3-BUTYRIC ACID-RESPONSE5 (IBR5) as a Novel Regulator of Organ Size in Arabidopsis
Source: PLoS One. 2015 Jul 6;10(7):e0131103. doi: 10.1371/journal.pone.0131103 (PMC4492785; doi:10.1371/journal.pone.0131103)

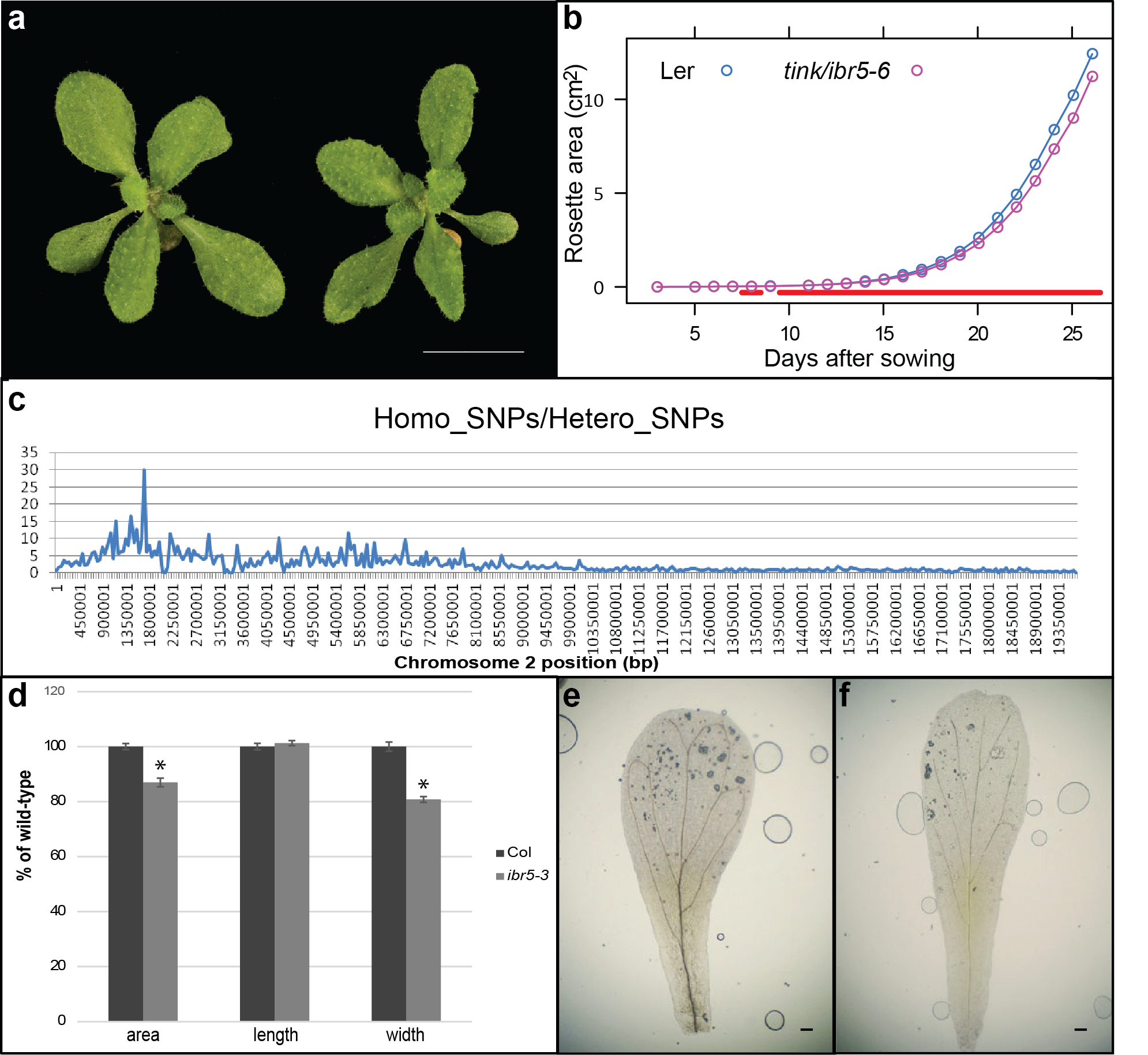

Supplement: S1 Fig — a. Representative Ler (left) and tink/ibr5-6 mutant (right) plants show the rosette leaves of tink/ibr5-6 mutants are narrower than wild-type. b. Kinematic analysis of tink1/ibr5-6 and Ler rosette size during development show tink1/ibr5-6 has significantly smaller rosette area (shown by red bar, P-values below 0.05 using Wilcoxon Rank Sum tests) c. SNP distribution on chromosome 2 in a mapping population of tink/ibr5-6 (Ler) crossed to Columbia. The ratio of homozygous SNPs to heterozygous SNPs was plotted (y-axis). d. Measurement of ibr5-3 and Col petal size shows a statistically significant (shown by *) reduction in petal area (p value ≤ 3e-7) and petal width (p value ≤ 1e-14) in ibr5-3 mutants using two tailed t-tests assuming unequal variance. Petals of Ler (e) and tink/ibr5-6 (f) mutants show defects in vein patterning in tink/ibr5-6 compared to wild-type. Scale bar is 1 cm in (a) and 0.1 mm in (e,f). (TIF) [file pone.0131103.s001.tif]

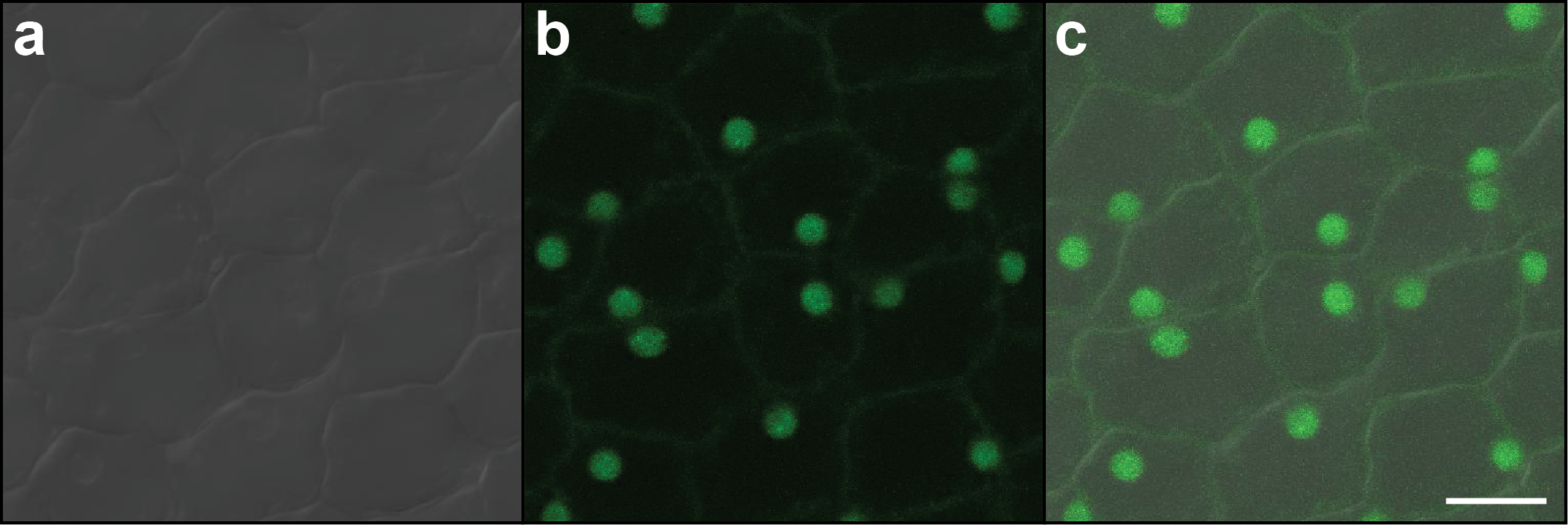

Supplement: S2 Fig — GFP is located in the nucleus, DIC (a), GFP fluorescence signal (b), and merge (c). Scale bar is 10μm. (TIF) [file pone.0131103.s002.tif]

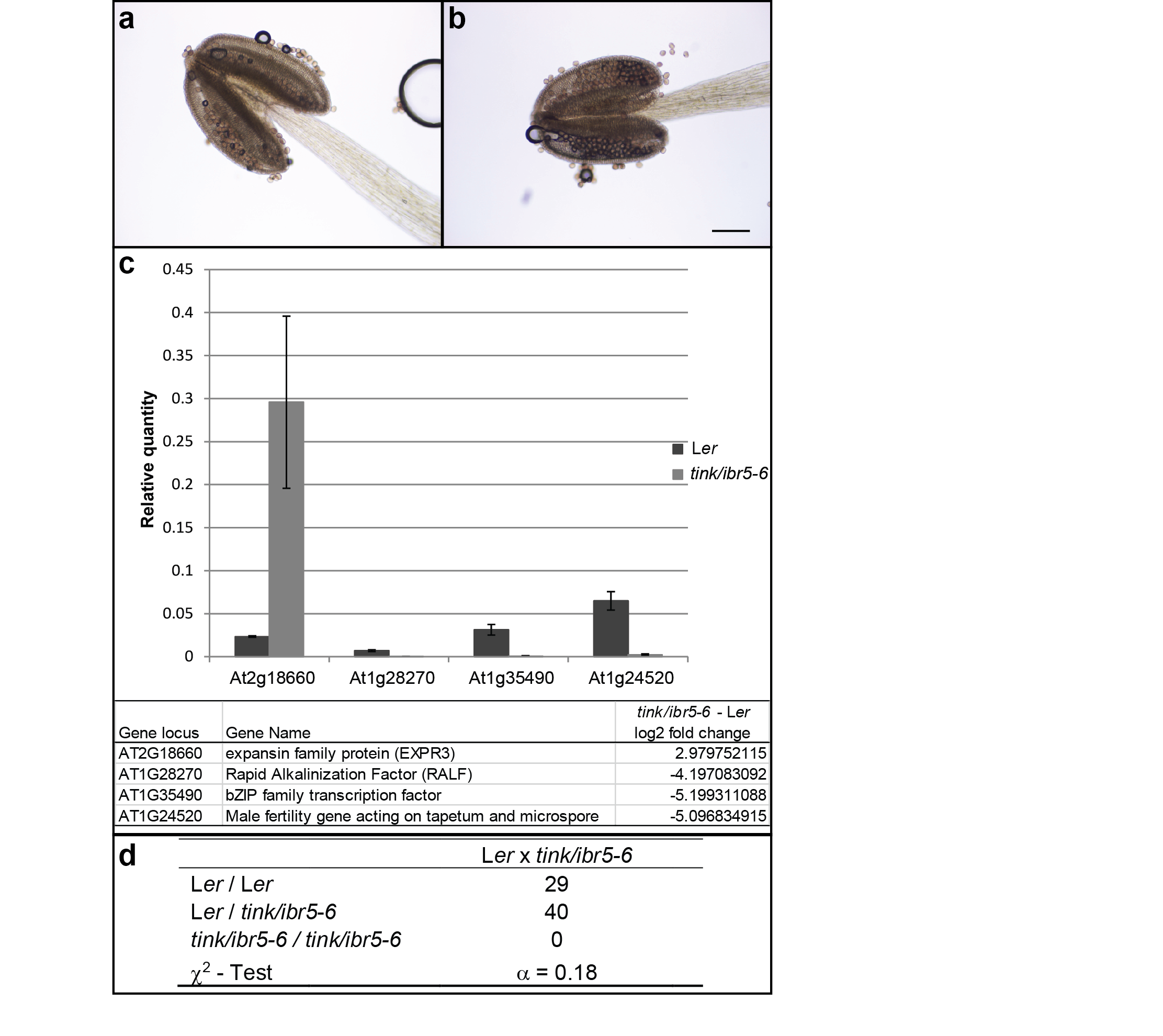

Supplement: S3 Fig — Phenotype of wild-type (Ler) (a) and tink/ibr5-6 (b) anthers show no obvious differences. c. Q-PCR (upper panel) and microarray (lower panel) analysis of genes specifically expressed in male gametophyte development, with altered expression in tink/ibr5-6 mutants compared to wild-type (Ler). d. Transmission efficiency of the tink/ibr5-6 mutation through male gametes. Scale in (a) and (b) is 0.1 mm. Values in (c) are shown as mean ± SEM from 3 biological and 3 technical replicates with expression levels normalized to that of the TUB6 gene. (TIF) [file pone.0131103.s003.tif]

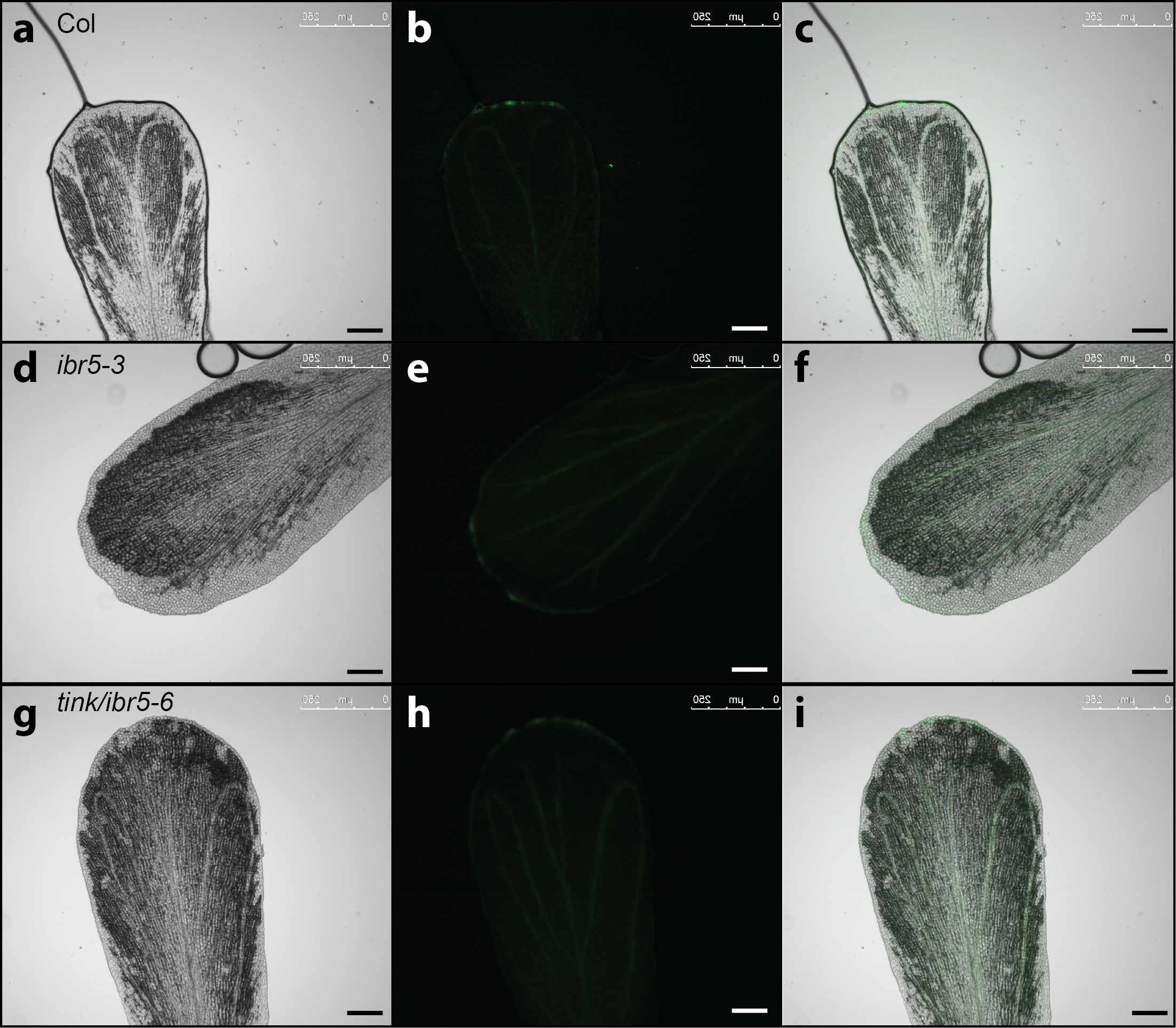

Supplement: S4 Fig — Bright-field image is shown in a, d and g, GFP fluorescence is shown in b, e and h and merged image is shown in c, f and i. Scale bar is 100 μm. (TIF) [file pone.0131103.s004.tif]

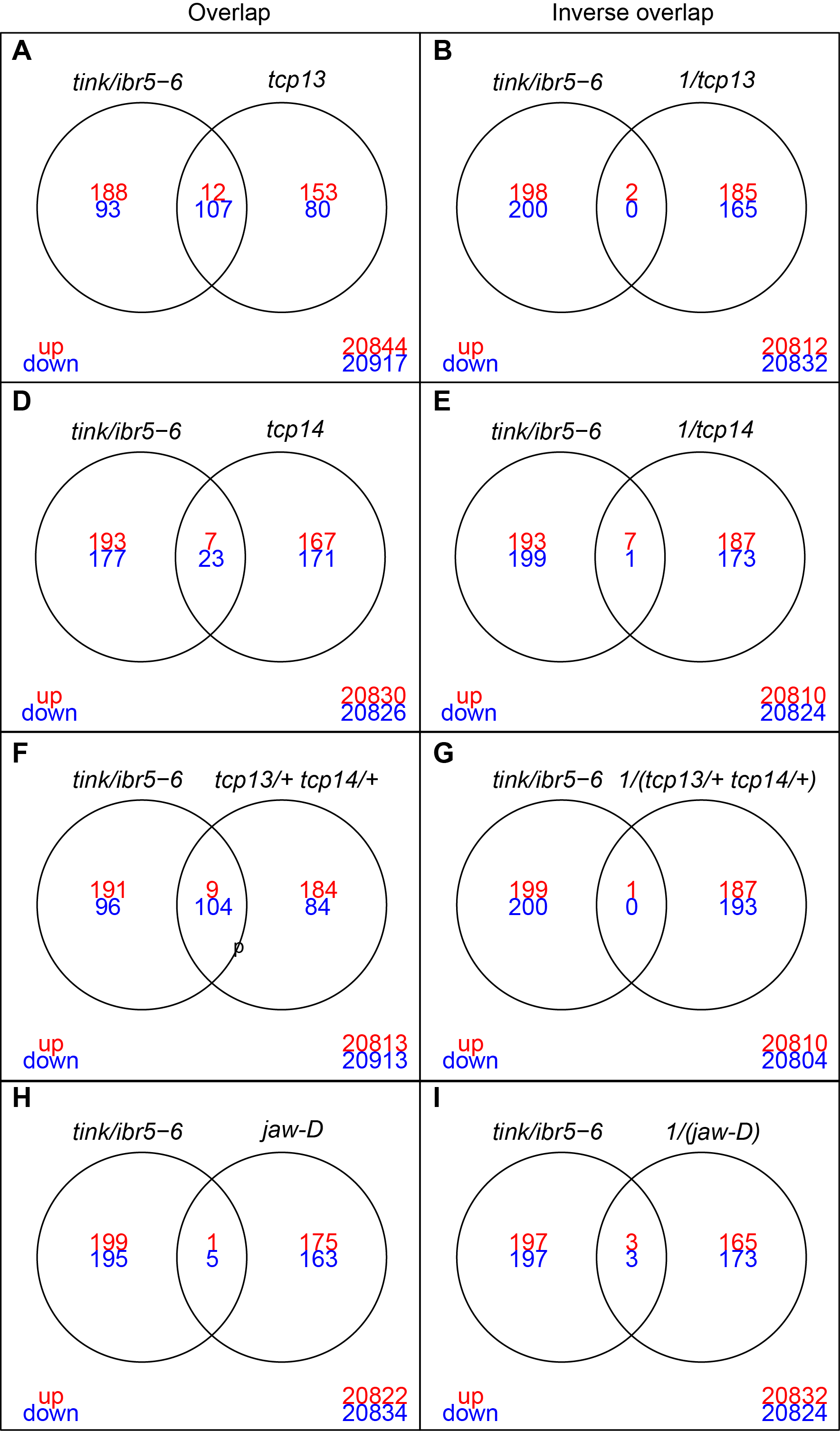

Supplement: S5 Fig — The number of overlapping genes (A, C, E and G) and the inverse overlap (B, D, F, H) significantly up (red) or down (blue) regulated is shown for comparison of tink/ibr5-6 with tcp13 (A, B), tcp14 (C, D), tcp13/+ tcp14/+ (E, F) and jaw-D (G, H). (TIF) [file pone.0131103.s005.tif]
