# Supplementary material for: The Tinkerbell (Tink) Mutation Identifies the Dual-Specificity MAPK Phosphatase INDOLE-3-BUTYRIC ACID-RESPONSE5 (IBR5) as a Novel Regulator of Organ Size in Arabidopsis
Source: PLoS One. 2015 Jul 6;10(7):e0131103. doi: 10.1371/journal.pone.0131103 (PMC4492785; doi:10.1371/journal.pone.0131103)
Supplement: S1 File — (DOCX) [file pone.0131103.s006.docx]

**Supporting Information**

**Supporting Experimental Procedures**

**Rosette growth measurements**

Plant growth was measured by taking whole rosette pictures from the top using a LemnaTec machine ([Arvidsson *et al.* 2011](#_ENREF_1)). Measurements were stopped at day 26 after sowing out as leaves started growing out of the pots. In total 140 plants were monitored, 84 *tink/ibr5-6* mutants and 56 L*er* controls. Rosette areas were segmented using Matlab with its Image Processing Toolbox. 12 *tink/ibr5-6* and 6 L*er* did not grow normally and were filtered out from the following analyses. Rosette areas for plants from both genotypes were compared using a Wilcoxon Rank Sum test at each day after sowing. P-values below 0.05 were considered significant. Average area values per genotype were plotted.

**Confocal Microscopy**

Imaging of petals expressing *pDR5*:GFP and *p35S*:GFP:IBR5 was performed using a Zeiss LSM 510 Meta confocal microscope**.**

**Transmission efficiency of gametes**

The gametophytic transmission of the *tink/ibr5-6* allele was determined as outlined in ([Howden *et al.* 1998](#_ENREF_2)). Crosses were performed between wild type (L*er*) and pollen of heterozygous *tink/ibr5-6* lines. The transmission efficiency through each gamete was determined using KASP genotyping of the *tink/ibr5-6* mutation in the progeny.

**Supporting references**

**Arvidsson, S., Perez-Rodriguez, P. and Mueller-Roeber, B.** (2011) A growth phenotyping pipeline for Arabidopsis thaliana integrating image analysis and rosette area modeling for robust quantification of genotype effects. *The New phytologist*, **191**, 895-907.

**Howden, R., Park, S.K., Moore, J.M., Orme, J., Grossniklaus, U. and Twell, D.** (1998) Selection of T-DNA-tagged male and female gametophytic mutants by segregation distortion in Arabidopsis. *Genetics*, **149**, 621-631.
